# Society Proceedings

**Published:** 1912-11

**Authors:** 


### SOCIETY PROCEEDINGS.

---

The Medical Society of the County of Wyoming met at Warsaw, Oct. 8. Officers were elected as follows: Dr. George H. Peddle, Perry, President; Dr. W. J. French, Pike. V. P.; Dr. L. Humphrey, Silver Springs, Sec.-Treas.

---

The Gross Medical Club met at the Park Club, Buffalo, Sept. 27, being entertained by Dr. A. E. Diehl of Buffalo. Dr.

F. A. Helwig of Akron, Health Officer of the town of Newstead, read a paper on Infantile Paralysis. The next meeting will be entertained by Dr. James E. King of Buffalo, late in October.

---

The Elmira Academy of Medicine meets in the Federation Building, at 8:15 P. M., on the first Tuesday of each month. At the October meeting, Dr. Ross C. Loop read a paper on the Bacillus Coli. At the November meeting, to be held Wednesday, Nov. 6, on account of election, Prof. V. A. Moore of Ithaca will read on Tetanus and Dr. Henry Flood will present some Reminiscences.

---

The Rochester Academy of Medicine usually meets on Wednesday evenings at 8:30. On Oct. 16, Section II. (Surgery and Surgical Specialities) met at the Rochester Club, holding a dinner in honor of the guest, Dr. George W. Crile of Cleveland, who later discussed the Tyroid Question.

---

The Buffalo Academy of Medicine meets on Tuesday evenings in the Public Library Building, at 8:30, there being usually a rotation of the sections, Surgery, Medicine, Obstetrics and Gynaecology, Pathology. With the exception of election night, there will be a meeting every Tuesday from Oct. 1 to May 20. Stated meetings of the entire Academy (which do not differ in general interest nor attendance from the section meetings, but at which the President of the Academy presides, if present, instead of the Section Chairman, business matters are considered, and a collation is served, will be held Oct. 15, Nov. 12, Jan. 7, and March 25. The annual meeting for the election of officers, annual report, etc., will be held June 10.

These general announcements are made as the Academies welcome guests, who should make themselves known to some member, be presented to the meeting and receive the privilege of the floor. We also suggest that physicians able to attend Academy meetings often, apply for non-resident membership.

On Oct. 1, at the Buffalo Academy of Medicine, Dr. Hideyo Noguchi of New York City, described the Life Cycle of the Treponema palidum and discussed spiral organisms generally. He has promised to publish the gist of his remarks in this Journal, later. Dr. Roswell Park read a paper on Isopathy in the Twentieth Century, based on Trade lists of organs, materies morbi, &c., still published and apparently indicating an actual persistence in their use.

On Oct. 8, Dr. Louis J. Hirschman of Detroit showed lantern slides illustrating the diagnosis of chronic colitis and its attendant dilatations, strictures and ptoses and discussed the effects of typhlostomy, resection and various other surgical procedures.

On Oct. 15 (Stated Meeting) there was a symposium on Accouchement Force: Vaginal Hysterectomy, Dr. W. H. Mansperger; Hebosteotomy, Dr. F. C. Goldsborough; Version and High Forceps, Dr. P. W. Peyma.

(Note: JOURNAL matter goes to printer about the 20th of each month and, as we dislike to make statements in pluperfect conditional, meetings and other events occurring after that date are left for the subsequent issue.)

---

THE MEDICAL SOCIETY OF THE COUNTY OF MONROE held its regular October meeting Oct. 22, at the rooms of the Rochester Academy of Medicine. Program: "Specialism: Medical-Legal and Expert Testimony." Dr. C .W. Hennington; "Hemorrhage in the New-Born; its Treatment and Report of Case," Dr. Norris G. Orchard; "Remarks on Physicians' Liability Insurance," Dr. James B. Woodruff. Dr. S. W. Little, President; Dr. Albert C. Snell, Secretary.

---

THE HOSPITAL MEDICAL SOCIETY OF ROCHESTER meets semi-monthly, October to May.The honary members number 40, the active about 30. On Oct. 10 Dr. George M. Gelser read a paper on the Treatment of Pueperal Infections; on Oct. 25, Dr. Bradford A. Richardson on Deafmutism. The officers are as follows: President, Myron B. Palmer; Vice-President, William H. Sutherland; Secretary and Treasurer, George H. Gage; Trustees, Richard M. Moore, Albert C. Snell, Norris G. Orchard, Frank P. Leadley, Ralph R. Fitch; Committee on Entertainment, Chairman, The Secretary; Willis E. Bowen, Bradford A. Richards.

---

THE CLINICAL CONGRESS OF SURGEONS OF NORTH AMERICA will meet in New York, Nov. 11-16, the Waldorf-Astoria being designated as Headquarters. Clinics will be held daily, from 8 to 5 and evening meetings will be held at which eminent surgeons of America and Europe will discuss "live surgical subjects." A general invitation to attend is issued to the profession. Visitors should register promptly at headquarters, pay the registration fee of $5.00 and receive detailed programs. The officers are as follows: Albert J. Ochsner, President; Edward Martin, President-Elect; John G. Clark. Vice-President; George E. Brewer, Vice-President-Elect; Franklin H. Martin, General Secretary; Allen B. Kanavel, Treasurer; A. D. Ballou, General Manager.

---

On the evening of October 2, 1912, there assembled at the International Hotel, Niagara Falls, as distinguished a body of German Scientists as it has been the honor of Americans to entertain. These gentlemen, numbering about 300, comprise most of the delegates to the International Congress of Demography, which has had its session at Washington, D. C.

Among their number were the members of the German Central Committee. This committee or association merits a description. It has for its object medical travel-study. Originated by a French physician, Dr. Carron de la Carriere, in Paris in 1899, the movement was readily and rapidly taken up by the German medical fraternity and now numbers about 700 members. Every year a trip is taken, the itinerary of which is so arranged that medical conventions may be included, the expenses curtailed as much as possible, and provisions made for entertainment and sight-seeing.

In Europe, it will be remembered, a craftsman, after having served his apprenticeship, is not considered to have his education completely rounded out until he has done a certain amount of traveling. Often such an *ausgelerter* will travel afoot from place to place for months, working here and there, that he may get the benefit of travel and diverse methods. As a matter of fact Europeans are much fonder of travel than are we and hold it in higher esteem as a means of education. They are thus much better acquainted by actual experience with their country, its resources, inhabitants, etc., than are we with ours. No one extracts so much culture and learning from travel as does the educated man. It is to be regretted that its indulgence is expensive and thereby denied to many younger members of our profession.

These ideas have been imbued, to a certain extent, in the object of the Central Committee. Their work has now been broadened to include study of social, economic, philanthorpic, hygienic, and civil institutions; factories, laboratories, mining plants, etc. This work being a post graduate course of a character highly useful to medical men, it would seem that such a work might well be taken up by our county societies.

The arrangements for the day's sight seeing and evening's entertainment of the guests on October 2d, were in the hands of Dr. Carl G. Leo-Wolf of Niagara Falls, to whom a great deal of credit is due. He was assisted in wlecoming the guests by the following medical men: Drs. Roswell Park, Lucien Howe, Henry Buswell, Edmond Blaauw, Max Breuer, William Gaertner, Julius Richter, A. W. Hengerer, James Putnam, Rolland Meisenbach, Herman Hayd, Peter VanPeyma, John Ragone, and Fridolin Thoma, all of Buffalo; Drs. J. S. Gianfranceschi, W. H. Hodges, W. D. Hough, A. J. Lawler, N. W. Price, W. A. Scott, Geo. D. Stillson, E. L. Burhyt, T. J. McBlain, W. L. Wilson, of Niagara Falls; Dr. C. N. Palmer, of Lockport, and Drs. W. E. Olmstead and F. W. Wilson, of Niagara Falls, Ontario.

The entertainment was in the form of a *kommers,* or student's convivial gathering, with Professor His of Berlin as toast master. After welcoming speeches by Dr. Gaertner, Dr. Howe and Dr. Blaauw, toasts were responded to by representatives of the various German Universities. A delegation of about thirty members from the Buffalo Orpheus delighted the visitors with German songs.

Among the more noted foreign gentlemen were Prof. Loefler, of the University of Greifswald; Prof. Weber, of the Imperial Health Bureau; Prof. Vossins, of the University of Giessen; Prof. Simon de Unterberger, privy councilor and honorary physician to his majesty's court of St. Petersburg, and Prof. His, professor of physiology at the University of Berlin.

225 of these German physicians sailed for home on the Victoria Louise, from New York, Oct. 10.

---

The Rochester Pathological Society held two meetings during October. At the first Dr. C. W. Hennington gave a paper on "Early Medical Annals of Rochester." The second meeting consisted of a symposium on "Proprietary Medicines," by Drs. C. R. Barber, J. M. Swan, and M. B. Palmer.

---

The Rochester Academy of Medicine held a meeting on October 16, 1912, at which Dr. George W. Crile of Cleveland spoke of "The Thyroid Question." A short time ago Lieut. Frost of the U. S. P. H. & M. H. Service spoke on " "Infantile Paralysis."

---

The Buffalo Medical and Surgical League had its regular monthly meeting on Thursday, October 10th, at the Markeen Hotel. The paper of the evening, entitled: "A Simplified Technique in House Operations," was read by Dr. Julius Richter. Several interesting cases were reported by members. An enjoyable lunch was served.

---

The 21st annual meeting of the ASSOCIATION OE ERIE RAILROAD SURGEONS was held at the New Sherman Hotel, Chicago, Oct. 8-11. About 90 Erie surgeons are members of this association, beside about 25 who do not belong to it. In addition to an interesting program of papers, clinics were held at various Chicago hospitals.

Officers of the Association, 1911-1912: President, H. F. Gillette, M. D., Cuba, N. Y.; Vice-President, S. Heilman, M. D., Sharon, Pa.; Secretary-Treasurer, B. R. Wakeman, M. D., Hornell, N. Y.; Executive Committee, H. P. Jack, M. D., Hornell,

N. Y.; W. J. Lowery, M. D., Carbondale, Pa.; T. C. Park, M. D. Akron, Ohio.

---

The 95th semi-annual meeting of the MEDICAL SOCIETY OF THE COUNTY OF STEUBEN was held at Hornell, Oct. 8.

---

THE CHAUTAUQUA COUNTY MEDICAL ASSOCIATION met at Westfield, Oct. 16. Dr. William S. Bainbridge of New York spoke on cancer. Dr. G. L. Hunter of Westfield and Dr. Fred Rice of Ripley also presented papers. The officers are: Dr. H. A. Eastman of Jamestown, President; Dr. N. G. Richmond of Fredonia, Vice-President; Dr. George F. Smith of Falconer, Second Vice-President, and Dr. W. J. Morris of Jamestown, Secretary and Treasurer. The Censors are Dr. E. M. Schofield of Jamestown, Dr. V. M. Griswold of Fredonia, and Dr. A. A. Becker, of Jamestown.

---

The Medico-Legal Society held its opening Fall Meeting at the Waldorf-Astoria on Wednesday, October 23rd, 1912, for the discussion of "Medical Expert Testimony in Homicidal Cases Where Insanity is Pleaded," and the merits of the Bill proposed by ex-Chief Justice Emery known as the Maine Bill.

1. Paper by Clark Bell, entitled "Medical Expert Testimony," and the merits of the Maine Bill.

2. Replies by ex- and Chief Justices Hon. Simeon E. Baldwin, of Connecticut; Hon. Orrin N. Carter, of Illinois; Hon. Charles M. Start, of Minnesota; Hon. Charles E. Frick, of Utah; Hon. William T. Spear, of Ohio; Hon. John R. Rowell, of Vermont; Hon. Eugene B. Gary, of South Carolina, and other Chief Justices of the Supreme Courts.

3. Views of Dr. R. L. Parsons of New York; Clarence A. Lightner, Esq., of Detroit, Michigan; Charles E. George, Esq., of San Francisco; Dr. E. E. S. McKee, of Cincinnati, Ohio, and other prominent alienists and medico-legal jurists.

4. The American Institute of Criminal Law and Criminology; the National State and City Bar Associations; the State Medical Societies, and all cognate associations are invited to send delegates to the discussion, and their members to contribute to the discussion, and the literature will be sent upon application.

5. Historical Sketch of the Supreme Court of Oregon, by Judge J. C. Moreland, of Salem, Oregon; The Supreme Court of West Virginia, by William B. Matthews, of Charleston, West Virginia.

6. The Chief Justices of the Supreme Courts of the States, by the Editor.

T. D. Crothers, President; Clark Bell, Secretary.

The Medical Society of the County of Erie held its regular meeting at the Buffalo Library Building on October 21st, 1912.

Two sessions were held—the first at 4.15 p. m. and the second at 8.30 p. m., the former being devoted to business and the latter to a scientific program. President Dr. Thomas H. McKee presided at both.

Secretary Gram read the minutes of the last meeting which was held June 17th, 1912, also the minutes of the Council meetings of October 7th and October 21st, 1912, all of which were duly approved.

On account of removal from the city, Drs. F. Frisch and R. A. Edson tendered their resignations which were accepted.

Dr. Charles A. Wall, Chairman of the Committee on Membership, presented applications for membership and applicants were elected as follows:

Harold J. McDonald, George J. Saylin, James E. Short, William F. Gallivan, Wellington M. Ross, Leon H. Prior, Timothy F. Donovan, Rose Rudolph Donk, Roy S. Moore, James C. Sullivan, Charles Leone and Jay Thornton Barnsdall.

Dr. Hopkins, Chairman of the Committee on Public Health, presented a report for his committee and submitted a draft of a letter addressed to the District Attorney in reference to school ventilation which, on motion of Dr. Wall, was approved and the committee directed to refer the matter to the District Attorney.

Dr. Bonnar, Chairman of the Board of Censors, submitted a lengthy report, on behalf of the Board, in which he stated that Matthew Stark, a barber of Lackawanna, N. Y., had been fined $25 for cupping; another man named Reeves was fined $50 for illegally practicing medicine in the office of Dr. Hughson, No. 6 South Division Street; $50 also had been recovered in the Treskow case, the fine having been imposed as far back as 1910; the board was also instrumental in bringing about several other indictments for criminal mal-practice. Dr. Bonnar stated that attorney Charles A. Doane, who, for several years, had acted as counsel for the society, had tendered his resignatioin and that attorney Alfred L. Harrison had been appointed in his stead. Report was adopted and the thanks of the society extended to the Board of Censors.

Dr. William H. Thornton, Chairman of the special committee on collection of accounts, reported progress and asked for further time which, on motion, was granted.

On motion of Dr. Lytle, the details for the conduct of the annual election was referred to the Council with power.

Dr. Woodruff brought up the question of pure water for the city, and after some discussion in which several members participated, it was moved by Dr. Thornton that Dr. Henry R.

Hopkins, Chairman of the Committee on Public Health, be elected to attend the joint meeting of the Great Lakes International Pure Water Association and the National Association for Preventing the Pollution of Rivers and Waterways to be held in Cleveland, Ohio, October 23rd and 24th, 1912, at the expense of the Society. Motion was carried.

President McKee then called for nominations for the various offices to be filled by election at the annual meeting to be held in Dcember, and nominations were entertained as follows:

For President, Dr. J. F. Whitwell; for Vice President, Dr. John V. Woodruff; Second Vice President, Drs. Arthur W. Hurd and Franklin W. Barrows; Secretary, Dr. Franklin C. Gram; Treasurer, Dr. Albert T. Lytle. Members of the Board of Censors, Drs. Bonnar, Fronczak, Bennett, Irving W. Potter, Hendee and A. D. Carpenter. Chairman, Committee on Legislation, Dr. F. Park Lewis; chairman, Committee on Public Health, Dr. Henry R. Hopkins; chairman, Committee on Membership, Drs. George J. Eckel and Harry Mead. Delegates to the State Society, of which five are to be elected—Drs. William H. Thornton, Arthur C. Schaefer, Charles A. Wall, S. A. Dunham, Thomas H. McKee, E. L. Frost, J. V. Woodruff, B. Cohen, George F. Cott and Cyrus S. Siegfried.

At the evening session, Dr. Hartwig read a memorial on the death of Dr. Ludwig Schroeter. On motion of Dr. Crego, the president was empowered to appoint fifty delegates to represent the society at the meeting of the Central New York Medical Association to be held in Batavia, N. Y., October 24th, 1912.

Dr. D. C. McKenney read a carefully prepared paper on "Some Considerations in the Treatment of Constipation."

Dr. James E. King presented an interesting paper on "Gynecology of Accident and Injury."

Dr. Roswell Park read a carefully prepared and very interesting paper, illustrated with lantern slides, on "The Pituitary Body."

Each of the papers elicited interesting discussion at the close of which a fine collation was served.

FRANKLIN C. GRAM, M. D.,
Secretary.

---

The Medical Association of Central New York held its annual meeting at Batavia, Oct. 24. An interesting program was provided. Luncheon was served to about 75 who attended the meeting. Several members remained to supper at the Holland Club, as guests of the President, Dr. Wm. D. Johnson, of Batavia. The following officers were elected for the ensuing year:

President, Dr. L. F. O'Neil of Auburn; Vice-Presidents, Drs. George M. Price of Syracuse and Floyd S. Crego of Buffalo; Secretary, Dr. John J. Buettner of Syracuse; Treasurer, Dr. C. O. Boswell of Rochester. The next meeting will be at Auburn.

---

The American Association of Clinical Research will hold its fourth annual meeting at 17 W. 43d St., New York City, Nov. 9, in Dubois Hall, N. Y. Academy of Medicine. The president is Dr. Alvin Roy Peebles of Boulder, Col.; the Secretary, Dr. James Krauss of Boston, to whom application for membership may be made.

